# The effects of conjugated linoleic acid supplementation on inflammatory cytokines and adipokines in adults: A GRADE-assessed systematic review and dose–response meta-analysis

**DOI:** 10.3389/fimmu.2023.1092077

**Published:** 2023-02-22

**Authors:** Samira Rastgoo, Ghazaleh Shimi, Farideh Shiraseb, Ashkan Karbasi, Damoon Ashtary-Larky, Mohsen Yousefi, Elnaz Golalipour, Omid Asbaghi, Mohammad Zamani

**Affiliations:** ^1^ Department of Cellular and Molecular Nutrition, Faculty of Nutrition Science and Food Technology, National Nutrition and Food Technology Research Institute, Shahid Beheshti University of Medical Sciences, Tehran, Iran; ^2^ Research Committee, Shahid Beheshti University of Medical Sciences, Tehran, Iran; ^3^ Department of Community Nutrition, School of Nutritional Sciences and Dietetics, Tehran University of Medical Sciences (TUMS), Tehran, Iran; ^4^ Department of Clinical Biochemistry, School of Medicine, Hamadan University of Medical Sciences, Hamadan, Iran; ^5^ Nutrition and Metabolic Diseases Research Center, Ahvaz Jundishapur University of Medical Sciences, Ahvaz, Iran; ^6^ Faculty of Medicine, Shahid Beheshti University of Medical Sciences, Tehran, Iran; ^7^ Cancer Research Center, Shahid Beheshti University of Medical Sciences, Tehran, Iran; ^8^ Department of Clinical Nutrition, School of Nutritional Sciences and Dietetics, Tehran University of Medical Sciences, Tehran, Iran

**Keywords:** inflammation, cytokines, adipokines, conjugated linoleic acid, meta-analysis

## Abstract

**Background and aims:**

Many studies have investigated the effect of conjugated linoleic acid (CLA) supplementation on inflammatory cytokines and adipokines. However, the results of these studies are not consistent. Therefore, this systematic review and meta-analysis were designed to comprehensively evaluate the effect of CLA supplementation on inflammatory cytokines and adipokines.

**Methods:**

Randomized controlled trials (RCTs) examining the effects of CLA supplementation on C-reactive protein (CRP), interleukin 6 (IL-6), tumor necrosis factor-alpha (TNF-α), adiponectin, and leptin, published up to March 2022, were identified through PubMed, SCOPUS, and ISI Web of Science databases. A random-effects model was used to calculate weighted mean differences (WMDs) with 95% confidence intervals (CI) for 42 studies that included 1,109 participants.

**Results:**

Findings from 42 studies with 58 arms indicated that CLA supplementation significantly decreased IL-6 and TNF-α levels and also slightly increased CRP levels. However, adiponectin and leptin levels did not change after CLA supplementation. A subgroup analysis found that CLA supplementation reduced adiponectin and leptin in women.

**Conclusion:**

Our results demonstrated that CLA supplementation increased CRP levels and decreased TNF-α and IL-6 levels. Therefore, it seems that CLA can have both proinflammatory and anti-inflammatory roles.

**Systematic review registration:**

https://www.crd.york.ac.uk/prospero/, identifier (CRD42022331110).

## Introduction

Inflammation is a protective reaction by an organism in response to injury, irritation, or infection that eliminates harmful stimuli and initiates the healing process (1). However, uncontrolled or unresolved inflammation can lead to tissue damage as well as the development of chronic inflammatory diseases, including type 2 diabetes, cardiovascular disease, cancer, etc. (2, 3). Therefore, the control and prevention of chronic inflammation should be considered in order to prevent some chronic diseases and even improve health.

Weight loss strategies such as dietary interventions and physical activity are commonly used as nonpharmacological interventions to combat and/or prevent proinflammatory conditions (4, 5). Despite the considerable benefits of pharmacological therapies, these may exert undesirable side effects and may not be tolerated by some individuals. On the other hand, they may cause drug resistance (reduction of efficiency) and even toxicity (6). Several studies have suggested that dietary strategies, such as certain supplements, can modulate inflammation. For example, a clinical trial has reported that magnesium and vitamin E co-supplementation led to a significant decrease in C-reactive protein (CRP) and a significant increase in total antioxidant capacity levels (7–9). Another study has suggested that l-glutamine supplementation in the early period of COVID-19 infection may reduce inflammatory responses and boost the immune system (10). Therefore, it is practical to find nutraceuticals and natural compounds with anti-inflammatory effects that may serve as alternative therapies to pharmacological interventions.

Conjugated linoleic acid (CLA) is a collective term for geometric isomers of linoleic acid (C18:2, n-6). This polyunsaturated fatty acid has two double bonds separated by a methylene group. This conjugation of the double bond is generally in positions 9 and 11 or 10 and 12 and may be a cis or trans configuration. Depending on the position and geometry of the double bonds, several isomers of CLA have been identified, such as 9cis,11trans-CLA and 10trans,12cis-CLA (11, 12). Most of the beneficial properties of CLA are elicited by these two main isomers. For example, 10trans,12cis-CLA is involved in catabolic processes such as lipolysis and fat oxidation, whereas 9cis,11trans-CLA seems to be the active anabolic agent. In addition, based on the evidence, 10trans,12cis-CLA is thought to be anticarcinogenic, antiobesity, and antidiabetic, whereas 9cis,11trans-CLA is mainly anti-inflammatory (13).

CLA is formed via biohydrogenation by bacterial enzymes known as catalyst, present in the intestine microbiota of ruminant mammals; hence, CLA is mainly found in their flesh and milk (14). Nonetheless, it is present in foods only in finite amounts; therefore, commercialized CLA supplements have been provided to offer potential benefits, such as reduction of body weight and total fat mass, anticancer effects including reducing tumor growth (15, 16), reducing insulin resistance, lipid disorders, and oxidative stress, improving liver function in nonalcoholic fatty liver (17), and immunomodulatory effects (18–20).

A large number of studies have investigated the effect of CLA supplementation on inflammatory cytokines and adipokines. However, the results of these studies are contradictory. For instance, some studies have reported that CLA supplementation had no significant effect on leptin levels (21, 22), whereas others found a significant decrease in serum leptin during CLA supplementation (23, 24). CLA supplementation has even been shown to significantly increase serum leptin levels (25). Also, some studies have reported that CLA supplements lead to a decrease in adiponectin levels (26), while another study has reported that CLA supplements have little or no effect on adiponectin levels (27). However, Sneddon et al. (28) have reported that CLA supplementation was associated with a significant increase in serum adiponectin levels (12%) in young obese subjects. Furthermore, while one study has found that CLA supplementation may increase CRP and tumor necrosis factor-alpha (TNF-α) and marginally decrease interleukin-6 (IL-6), indicating a proinflammatory state (29, 30), a recent review article suggests that CLA may have an anti-inflammatory effect by reducing inflammatory mediators such as cytokines, particularly IL-6, TNF-α, IFN-γ, and IL-1β (30). Therefore, due to the inconsistencies in previous studies, the present study sought to update previous meta-analyses in light of the plethora of new studies. Thus, the current meta-analysis sought to investigate the effects of CLA on inflammatory cytokines and adipokines in adults.

## Method and materials

This systematic review has been conducted according to the PRISMA statement (31). The present study was registered at PROSPERO CRD42022331110.

### Search strategy

We conducted a comprehensive literature search in scientific databases such as PubMed/Medline, Scopus, Web of Science, EMBASE, the Cochrane databases, and Google Scholar to find relevant prospective studies on the effects of CLA supplementation on inflammatory cytokines, adiponectin, and leptin published up to March 2022. There were no restrictions on the length of time or language of publications. The Participant, Intervention, Comparison/Control, Outcome (PICOS) search framework was used (32), which stands for population (both healthy and unhealthy adults), intervention (CLA supplementation), comparison (placebo/control group), outcome (changes in CRP, IL-6, TNF, adiponectin, and leptin), and research design (parallel and crossover randomized clinical trials).

The combination of MESH and non-MESH terms were used for the search, as follows: (“conjugated linoleic acid” OR “conjugated fatty acid” OR “boric acid” OR “rumenic acid” OR “CLA”) AND (intervention OR “intervention study” OR “intervention studies” OR “controlled trial” OR randomized OR random OR randomly OR placebo OR “randomized controlled trial” OR “randomized clinical trial” OR RCT OR blinded OR “double blind” OR “double blinded” OR trial OR “clinical trial” OR trials OR “Pragmatic Clinical Trial” OR “Cross-Over Studies” OR “Cross-Over” OR “cross-over study” OR parallel OR “parallel study” OR “parallel trial”).

### Study selection

By reading the titles, abstracts, and full text of the papers as needed, two researchers independently (FS and OA) chose the appropriate articles. The effects of CLA supplementation on inflammatory cytokines and adipokines variables in adults with different health statuses in all human randomized clinical trials)RCTs(.

We incorporated studies that satisfied the following criteria: (1) RCTs (parallel or crossover); (2) used oral intake of CLA; (3) examined the effects of CLA supplementation on CRP, IL-6, TNF-α, adiponectin, and leptin; (4) had an intervention duration of at least 2 weeks (RCTs with two or more eligible arms were considered as separate studies); (5) were performed on adults (≥18 years old); (6) provided means and standard deviations (SDs) for all outcome variables, or any other effect sizes from which the calculation of mean and SD was possible. The searches were limited to human studies with no language restrictions. Animal studies, reviews, *in vitro* research, research on kids and teenagers, grey literature, conference abstracts, opinion pieces, books, and RCTs without a placebo or control group were excluded. Studies that used CLA in combination with vitamins or minerals were also excluded.

In the present study, we searched for studies that assessed the effects of CLA supplementation on all inflammatory cytokines. After screening and finding eligible studies, we found that most studies evaluated CRP, IL-6, and TNF. In addition, a limited number of studies have evaluated other inflammatory factors such as IL-1 and IL-8. Therefore, we included studies that evaluated the effects of CLA on CRP, IL-6, and TNF.

### Data extraction

After quickly skimming the titles, abstracts, and full text to choose the most pertinent research following a separate review of each qualifying RCT, the following data were gleaned by two independent researchers (OA and FS). Name of the first author, country of origin, year of publication, type of clinical trial, participant characteristics (mean age, body mass index (BMI), sex), randomization, blinding, sample size, number of participants in the intervention and control groups, type and dosage of supplemented CLA, duration of the study, and related data were extracted for additional measurements. The CLA dosages were converted to milligrams per day, whether they were given in grams per day or another unit.

### Quality assessment

To rate the quality of the studies, the Cochrane Collaboration technique was utilized (33). Two researchers (SR and GS) independently assessed the methods, and any disagreements between their assessments were resolved through discussion. Each study was evaluated for any bias, including those caused by randomized sequence generation, allocation concealment, participant and staff blindness, outcome assessor blindness, insufficient outcome data, selective reporting, and other biases. Thus, three categories were established: high risk of bias (general risk of bias > 2 high risks), low risk of bias (general risk of bias < 2 high risks), and undetermined risk of bias (general risk of bias = 2 high risk).

### Statistical analysis

Stata 11.0 was used to conduct the statistical analysis (Stata Corp, College Station, TX, USA). *p*-values <0.05 were deemed statistically significant for all two-tailed tests. The pooled weighted mean difference (WMD) was calculated using a random-effects model to take into account any existing heterogeneity (due to the different intervention doses, duration, participant health, sample sizes, and length of intervention) developed by Der Simonian and Laird (34). We computed the mean differences in CRP, IL-6, TNF-α, adiponectin, and leptin between the CLA supplementation and control groups from the preintervention to the postintervention. Formula: SD = square root [(SD at baseline)^2^ + (SD at end of study)^2^ (2rSD at baseline, SD at end of study)] was used to get the SD of the mean difference (35). For RTCs that only provided standard error of the mean (SEM) information, SD was determined using the following formula: Standard Error of Mean (SEM): SD =, where *n* is the number of subjects in each group (36). After visual inspection of forest plots or Cochrane’s *Q* test, heterogeneity was evaluated using the *I* square (*I* ^2^) statistic (*p* < 0.05 and *I* ^2^ > 40%), with *I* ^2^ > 40% considered clinically significant (37). The baseline CRP, IL-6, TNF-α, adiponectin, and leptin levels were analyzed, while the intervention duration (< 12 weeks, ≥ 12 weeks) and dosage of CLA (≥ 3 and < 3 g/day) were based on the median values of the included studies and health status (healthy: people without any disease; unhealthy: people suffering from a disease such as atherosclerosis, rheumatoid arthritis, diabetes, etc.). Other subgroup analyses were performed according to gender (man, woman), baseline BMI (normal (18.5–24.9 kg/m²), overweight (25–29.9 kg/m²), and obese (>30 kg/m²)) and isomer’s type (CLA-c9t11, CLA-t10c12, both). Studies examining the effect of CLA supplementation on inflammatory cytokines and adipokines used the Begg’s test and the funnel plot test to evaluate publication bias (38). We used the leave-one-out approach to do a sensitivity analysis to identify how many inferences were dependent on a single sample to examine the influence of each study on the pooled effect size (39). The possible linear association between CLA (mg/day) dose and duration on outcome variables was evaluated using meta-regression, whereas a nonlinear dose–response analysis was used to assess the effect of CLA supplementation dose and duration (40, 41).

## Result

### The flow of study selection

**Figure 1:**
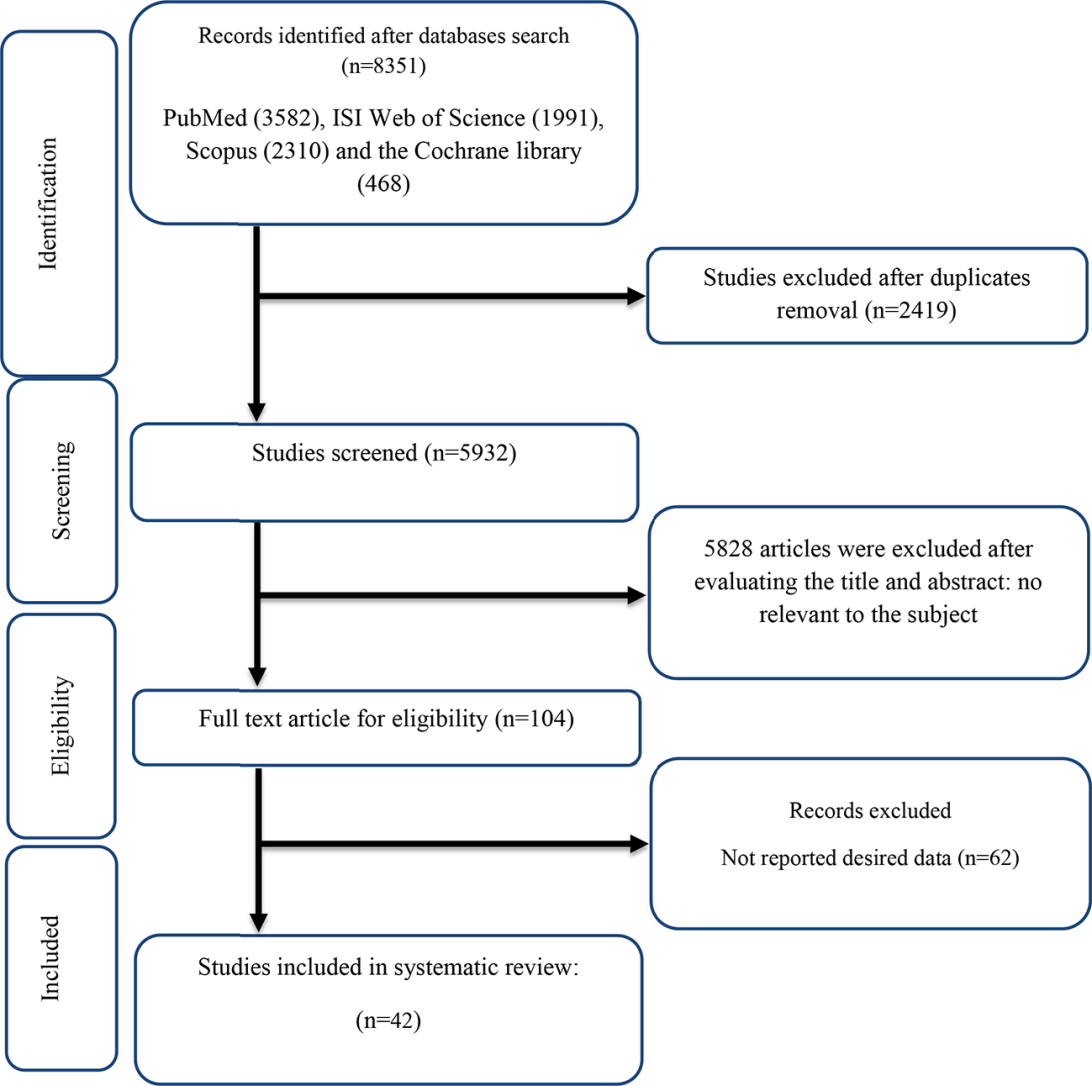
Flow chart of study selection for inclusion trials in the systematic review.

The flow chart presented in **Figure 1** describes the selection process and the references retrieved from the database.

A total of 8,351 studies were identified in the first step of the literature search of electronic databases, PubMed (*n* = 3,582), ISI Web of Science (*n* = 1,991), Scopus (*n* = 2,310), and the Cochrane library (*n* = 468). After excluding duplicated (*n* = 2,419) and irrelevant studies based on titles and abstracts (*n* = 2,804), 104 potentially relevant articles were considered for full-text review. Out of 104 studies, 62 did not have the desired data. Finally, 42 studies (11, 12, 16, 18, 19, 27, 28, 42–76) were included in the present meta-analysis, and their characteristics are illustrated in **Table 1**. The risk of bias assessment is summarized in **Table 2**.

**Table 1:** Characteristics of the included studies.

Studies	Country	Study design	Participant	Sex	Sample size		Trial duration (week)	Means age		Means BMI		Intervention		
					IG	CG		IG	CG	IG	CG	CLA dose	Isomer’s type	Control group Whigham
Medina et al. (42)	USA	Parallel, R, PC, B	Healthy	F: 17	10	7	9	20–41	20–41	23.2 ± 1.5	22.2 ± 3.9	3	22.6% 10trans,12cis; 23.6% 11cis,13trans; 17.6% 9cis,11trans; 16.6% 8trans,10cis; 7.7% 9trans,11trans and 10trans,12trans; and 11.9% other isomers	Placebo
Riserus et al. (43)	Sweden	Parallel, R, PC, DB	Obese men with the metabolic syndrome	M: 38	19	19	12	51 ± 7.1	53 ± 10.1	30.1 ± 1.8	30.2 ± 1.8	3.4	35.9% t10c12 CLA, 35.4% c9t11 CLA	Placebo
Riserus et al. (43)	Sweden	Parallel, R, PC, DB	Obese men with the metabolic syndrome	M: 38	19	19	12	55 ± 7.1	53 ± 10.1	31.2 ± 2.5	30.2 ± 1.8	3.4	CLA-t10c12	Placebo
Albers et al. (44)	Netherlands	Parallel, R, PC, DB	Healthy men	M: 47	25	22	12	52 ± 9	53 ± 10	25.3 ± 2.8	25.3 ± 2.3	1.7	50% c9,t11 CLA and 50% t10,c12	Control diet
Albers et al. (44)	Netherlands	Parallel, R, PC, DB	Healthy men	M: 46	24	22	12	52 ± 8	53 ± 10	25.7 ± 2.5	25.3 ± 2.3	1.6	80% c9,t11 CLA and 20% t10,c12	Control diet
Riserus et al. (45)	Sweden	Parallel, R, PC, DB	Obese men	M: 38	19	19	12	51 ± 7.1	53 ± 10.1	30.1 ± 1.8	30.2 ± 1.8	3.4	Both	Control diet
Riserus et al. (45)	Sweden	Parallel, R, PC, DB	Obese men	M: 38	19	19	12	55 ± 7.1	53 ± 10.1	31.2 ± 2.5	30.2 ± 1.8	3.4	CLA-t10c12	Control diet
Ramakers et al. (50)	France	Parallel, R, PC, DB	Overweight subjects	M/F (F: 13, M: 13)	14	12	13	53 ± 7	58 ± 5	29 ± 2	29 ± 2	3	CLA-c9t11	Control diet
Ramakers et al. (50)	France	Parallel, R, PC, DB	Overweight subjects	M/F (F: 12, M: 12)	12	12	13	56 ± 6	58 ± 5	29 ± 3	29 ± 2	3	CLA-t10c12	Control diet
Desroches et al. (46)	Canada	Crossover, R, PC, B	Overweight and obese	M: 17	17	17	4	36.6 ± 12.4	36.6 ± 12.4	31.2 ± 4.4	31.2 ± 4.4	4.22	Both	Control diet
Song et al. (48)	UK	Parallel, R, PC, DB	Young healthy volunteers	M/F (F: 20, M: 8)	14	14	12	31.8 ± 6.88	30.9 ± 7.14	24.3 ± 3.8	24.23 ± 3.69	3	50% c9,t11 CLA and 50% t10,c12	Control diet
Gaullier et al. (47)	Norway	Parallel, R, PC, DB	Healthy overweight humans	M/F (F: 74, M: 14)	47	41	104	48.6 ± 10.6	45.1 ± 8.8	28.3 ± 1.5	27.4 ± 1.7	3.4	50% c9,t11 CLA and 50% t10,c12	Placebo
Gaullier et al. (47)	Norway	Parallel, R, PC, DB	Healthy overweight humans	M/F (F: 69, M: 18)	46	41	104	45.1 ± 10.5	45.1 ± 8.8	28.1 ± 1.4	27.4 ± 1.7	3.4	50% c9,t11 CLA and 50% t10,c12	Placebo
Smedman et al. (49)	Sweden	Parallel, R, PC, DB	Healthy	M/F (F: 26, M: 27)	28	25	12	43.312.8	47.8 ± 10.1	25.8 ± 3.9	24.4 ± 4.2	4.2	50% c9,t11 CLA and 50% t10,c12	Control diet
Naumann et al. (52)	Netherlands	Parallel, R, PC, DB	Overweight subjects with LDL phenotype B	M/F: 68	34	34	13	51 ± 7	51 ± 9	28.6 ± 2.3	28 ± 2.2	3	CLA-c9t11	Control diet
Naumann et al. (52)	Netherlands	Parallel, R, PC, DB	Overweight subjects with LDL phenotype B	M/F: 53	19	34	13	55 ± 7	51 ± 9	29.3 ± 2.4	28 ± 2.2	3	CLA-t10c12	Control diet
Colakoglu et al. (11)	Turkey	Parallel, R, PC, SB	Healthy	F: 26	12	14	6	21.7 ± 2	20.4 ± 2.5	22.5 ± 1.7	21.6 ± 1.6	3.6	Both	Control diet–exercise
Colakoglu et al. (11)	Turkey	Parallel, R, PC, SB	Healthy	F: 18	11	7	6	20.4 ± 1.7	21.9 ± 2	23.3 ± 1.2	20.8 ± 1.6	3.6	Both	Control diet
Taylor et al. (51)	UK	Parallel, R, PC, DB	Healthy	M/F: 40	21	19	12	45 ± 6	47 ± 8	33 ± 3	33 ± 3	4.5	35% 9c,11t CLA, 36% t10,c12 CLA	Control diet
Mullen et al. (57)	Ireland	Parallel, R, PC, DB	Healthy middle-aged	M: 30	15	15	8	51 ± 8.04	47.8 ± 6.36	26.2 ± 2.32	26 ± 3.4	2.2	50% c9,t11 CLA and 50% t10,c12	Placebo
Nazare et al. (53)	France	Parallel, R, PC, DB	Healthy subjects	M/F: 44	21	23	14	29.4 ± 6.75	28.5 ± 5.7	25.2 ± 1.45	25.1 ± 1.48	3.76	50% c9,t11 CLA and 50% t10,c12	Placebo
Iwata et al. (55)	Japan	Parallel, R, PC, DB	Overweight	M: 40	20	20	12	44.3 ± 10.2	42.5 ± 10.4	27.4 ± 2	27.8 ± 1.9	5.4	50% c9,t11 CLA and 50% t10,c12	Placebo
Iwata et al. (55)	Japan	Parallel, R, PC, DB	Overweight	M: 40	20	20	12	40.5 ± 8.8	42.5 ± 10.4	28.1 ± 2.1	27.8 ± 1.9	10.8	50% c9,t11 CLA and 50% t10,c12	Placebo
Steck et al. (56)	UK	Parallel, R, PC, DB	Healthy obese humans	M/F (F: 23, M: 9)	16	16	12	36.3 ± 8.9	34.9 ± 8	32.7 ± 1.8	32.7 ± 1.9	3.2	50% c9,t11 CLA and 50% t10,c12	Placebo
Steck et al. (56)	UK	Parallel, R, PC, DB	Healthy obese humans	M/F (F: 24, M: 8)	16	16	12	34.1 ± 8.9	34.9 ± 8	32.7 ± 1.7	32.7 ± 1.9	6.4	50% c9,t11 CLA and 50% t10,c12	Placebo
Whigham et al. (54)	Canada	Parallel, R, PC, DB	Healthy	M/F (F: 32, M: 8)	22	18	24	34 ± 8	32 ± 7	27.6 ± 1.8	28 ± 2.2	3.2	39.2% 9cis,11 trans and 38.5% 10trans,12cis	Placebo
Gaullier et al. (27)	Norway	Parallel, R, PC, DB	Overweight and obese	M/F (F: 84, M: 21)	55	50	24	45.8 ± 10	48.7 ± 9.2	30.5 ± 10.4	30.2 ± 10.4	3.4	50% c9,t11 CLA and 50% t10,c12	Placebo
Raff et al. (61)	Denmark	Parallel, R, PC, DB	Healthy young men	M: 38	18	20	5	25.7 ± 4.2	26.1 ± 3.6	22 ± 1.9	22.5 ± 2.1	5.5	39.4% 9cis,11trans and 38.5% 10trans,12cis	Control diet
Aryaeian et al. (60)	Iran	Parallel, R, PC, DB	Rheumatoid Arthritis	M/F (F: 38, M: 6)	22	22	12	46.23 ± 13.07	47.95 ± 11.14	27.18 ± 0.99	28.48 ± 0.84	2.5	50% c9,t11 CLA and 50% t10,c12	Placebo
Kim et al. (58)	Korea	Parallel, R, PC, DB	Healthy overweight women	F: 27	15	12	12	26.33 ± 9.4	29.5 ± 10.8	25.23 ± 2.16	26.47 ± 1.8	3	50% c9,t11 CLA and 50% t10,c12	Control diet
Sneddon et al. (28)	UK	Crossover, R, PC, DB	Young lean	M: 13	13	13	12	30.5 ± 4.9	30.5 ± 4.9	23.6 ± 1.5	23.6 ± 1.5	3	50% c9,t11 CLA and 50% t10,c12	Placebo
Sneddon et al. (28)	UK	Crossover, R, PC, DB	Young obese	M: 12	12	12	12	32.4 ± 2.3	32.4 ± 2.4	32.3 ± 1.9	32.3 ± 1.9	3	50% c9,t11 CLA and 50% t10,c12	Placebo
Sneddon et al. (28)	UK	Crossover, R, PC, DB	Older lean	M: 20	20	20	12	56.3 ± 4.2	56.3 ± 4.2	23.6 ± 1.5	23.6 ± 1.5	3	50% c9,t11 CLA and 50% t10,c12	Placebo
Sneddon et al. (28)	UK	Crossover, R, PC, DB	Older obese	M: 14	14	14	12	56.9 ± 5.4	56.4 ± 5.4	32 ± 1.6	32 ± 1.6	3	50% c9,t11 CLA and 50% t10,c12	Placebo
Turpeinen et al. (59)	Finland	Parallel, R, PC	Subjects with birch pollen allergy	M/F (F: 28, M: 12)	20	20	12	20–46	20–46	NR	NR	2	65·3% 9cis,11trans-CLA and 8·5% 10trans,12cis-CLA	Placebo
Norris et al. (63)	Germany	Parallel, R, PC, DB	Postmenopausal women with type 2 diabetes mellitus	F: 55	22	33	16	59.4 ± 7.3	60.1 ± 7.3	37.1 ± 7.2	36.3 ± 6.1	6.4	41.6% c9,t11 CLA and 40.4% t10,c12	Control diet
Zhao et al. (62)	China	Parallel, R, PC, DB	Obesity-related hypertension	M/F (F: 36, M: 44)	40	40	8	62.3 ± 3.5	59.4 ± 2.4	32.3 ± 2.3	31.2 ± 1.4	4.5	50% c9,t11 CLA and 50% t10,c12	Control diet
Sluijs et al. (65)	Netherlands	Parallel, R, PC, DB	Overweight and obese adults	M/F (F: 179, M: 167)	173	173	24	58 ± 0.4	58.8 ± 0.5	28 ± 9.45	27.7 ± 12.75	4	CLA-c9t11	Placebo
Tavakkoli Darestani et al. (66)	Iran	Parallel, R, PC, DB	Postmenopausal	F: 76	38	38	12	55.1 ± 6.4	54.9 ± 6.9	27.6 ± 3.4	27 ± 3.4	3.2	50% c9,t11 CLA and 50% t10,c12	Placebo
MacRedmond et al. (12)	Canada	Parallel, R, PC, DB	Overweight mild asthmatics	M/F (F: 15, M: 13)	15	13	12	32.2 ± 8.7	29.9 ± 3.8	27.8 ± 4.5	27.3 ± 3.6	4.5	50% c9,t11 CLA and 50% t10,c12	Placebo
Sofi et al. (64)	Italy	Crossover, R, PC	Healthy middle-aged	M/F (F: 6, M: 4)	10	10	8	45.6	45.6	25 ± 4	25 ± 4	3	Both	Control diet
Joseph et al. (68)	Canada	Crossover, R, PC, DB	Overweight, hyperlipidemic	M: 27	27	27	8	18–60	18–60	31.5 ± 4	31.3 ± 4	3.5	50% c9,t11 CLA and 50% t10,c12	Placebo
Joseph et al. (68)	Canada	Crossover, R, PC, DB	Overweight, hyperlipidemic	M: 27	27	27	8	18–60	18–60	31.4 ± 4	31.3 ± 4	3.5	50% c9,t11 CLA and 50% t10,c12	Placebo
Pfeuffer et al. (69)	Germany	Parallel, R, PC, DB	Obese male subjects	M: 40	21	19	4	45–68	45–68	28.3 ± 2.3	27.8 ± 1.3	4.5	50% c9,t11 CLA and 50% t10,c12	Control diet
Brown et al. (67)	USA	Paralrell, R, PC	Health in young women	F: 18	9	9	8	20–40	20–40	19–30	19–30	1.17	Both	Control diet
Rubin et al. (70)	Germany	Crossover, R, PC, DB	Middle-aged men	M: 35	35	35	4	45–68	45–68	26 ± 2.6	26.1 ± 3	4.25	CLA-c9t11	Control diet
Rubin et al. (70)	Germany	Crossover, R, PC, DB	Middle-aged men	M: 35	35	35	4	45–68	45–68	26 ± 3.5	26.1 ± 3	4.25	CLA-t10c12	Control diet
Eftekhari et al. (73)	Iran	Parallel, R, PC	Atherosclerotic patients	M/F: 57	29	28	8	52.79 ± 14.11	55.85 ± 14.13	24.02 ± 2.76	24.66 ± 2.34	3	50% c9,t11 CLA and 50% t10,c12	Control diet
Shadman et al. (71)	Iran	Parallel, R, PC, DB	Overweight type2 diabetics	M/F (F: 21, M: 18)	19	20	8	45.1 ± 5.7	45.5 ± 4.3	27.4 ± 0.5	27.1 ± 1.8	3	50% c9,t11 CLA and 50% t10,c12	Placebo
Bulut et al. (72)	Turkey	Parallel, R, PC, DB	Young men	M: 18	9	9	4	19–31	19–31	27.5 ± 2.6	26.8 ± 1.9	3	50% c9,t11 CLA and 50% t10,c12	Placebo
Lopez-Plaza et al. (16)	Spain	Parallel, R, PC, DB	Healthy overweight people	M/F (F: 29, M: 9)	22	16	24	43 ± 8.3	44.35 ± 7.79	28.44 ± 1.08	28.56 ± 0.95	3	50% c9,t11 CLA and 50% t10,c12	Placebo
Aryaeian et al. (19)	Iran	Parallel, R, PC, DB	Rheumatoid arthritis	M/F (F: 38, M: 6)	22	22	12	46.23 ± 13.07	47.95 ± 11.14	27.18 ± 4.63	28.48 ± 3.94	2.5	50% c9,t11 CLA and 50% t10,c12	Placebo
Dus-Zuchowska et al. (75)	Poland	Parallel, R, PC, DB	Overweight and obese	F: 74	37	37	12	54 ± 4	54 ± 4	34 ± 3.5	35.36 ± 3.56	3	50% c9,t11 CLA and 50% t10,c12	Placebo
Baghi et al. (74)	Iran	Parallel, R, PC, DB	Athletic	M: 23	13	10	2	18.46 ± 1	18.2 ± 0.5	23.13 ± 0.89	23.83 ± 2.18	5.6	Both	Placebo
Abedi et al. (76)	Iran	Parallel, R, PC, SB	Nonalcoholic fatty liver disease	M/F (F: 32, M: 6)	19	19	8	36.74 ± 6.87	38.58 ± 8.24	32.72 ± 4.63	35.27 ± 3.46	3	Both	Control diet
Aslani et al. (18)	Iran	Parallel, R, PC, DB	Chronic obstructive pulmonary disease (COPD)	M/F: 82	40	42	6	63.82 ± 10.58	61.55 ± 10.81	25.46 ± 3.85	45.71 ± 26.11	3.2	Both	Placebo

**Table 2:** Risk of bias assessment.

Studies	Random sequence generation	Allocation concealment	Selective reporting	Other sources of bias	Blinding (participants and personnel)	Blinding (outcome assessment)	Incomplete outcome data	General risk of bias
Medina et al. (42)	L	H	H	H	U	H	L	Bad
RISerus et al. (43)	L	H	H	L	L	U	H	Bad
Albers et al. (44)	L	L	H	L	L	U	L	Good
Riserus et al. (45)	L	H	H	H	L	U	H	Bad
Desroches et al. (46)	L	H	H	H	L	U	L	Bad
Gaullier et al. (47)	L	H	L	L	L	U	L	Good
Song et al. (48)	L	H	L	L	L	U	L	Good
Smedman et al. (49)	L	H	H	L	H	H	L	Bad
Ramakers et al. (50)	L	L	H	L	L	L	L	Good
Colakoglu et al. (11)	L	H	H	H	L	U	H	Bad
Taylor et al. (51)	L	H	H	L	L	U	L	Fair
Naumann et al. (52)	L	H	H	H	H	H	H	Bad
Gaullier et al. (27)	L	L	L	H	L	U	H	Fair
Nazare et al. (53)	L	H	L	L	L	U	L	Good
Iwata et al. (55)	L	H	H	H	L	U	L	Bad
Watras et al. (54)	L	H	H	H	L	U	L	Bad
Mullen et al. (57)	L	H	H	H	L	U	L	Bad
Steck et al. (56)	L	H	H	L	L	U	L	Fair
Kim et al. (58)	L	H	H	H	H	H	L	Bad
Sneddon et al. (28)	L	H	H	L	L	U	L	Fair
Turpeinen et al. (59)	L	H	H	H	L	U	H	Bad
Aryaeian et al. (60)	L	H	H	H	L	U	H	Bad
Raff et al. (61)	L	H	H	H	L	U	H	Bad
Zhao et al. (62)	L	H	H	H	L	U	L	Bad
Norris et al. (63)	L	L	H	L	L	U	L	Good
MacRedmond et al. (12)	L	H	H	H	H	H	L	Bad
Tavakkoli Darestani et al. (66)	L	L	H	H	L	U	L	Fair
Sofi et al. (64)	L	L	H	L	L	U	L	Good
Sluijs et al. (65)	L	L	H	L	L	U	L	Good
Joseph et al. (68)	L	H	L	H	L	U	H	Bad
Brown et al. (67)	L	H	H	H	L	U	H	Bad
Pfeuffer et al. (69)	L	H	H	L	H	H	H	Bad
Rubin et al. (70)	L	L	H	H	L	U	L	Fair
Shadman et al. (71)	L	H	L	L	L	U	L	Good
Lopez-Plaza et al. (16)	L	L	H	H	L	U	L	Fair
Bulut et al. (72)	L	L	H	L	L	U	L	Good
Eftekhari et al. (73)	L	H	H	H	L	U	L	Bad
Aryaeian et al. (19)	L	H	H	H	L	U	L	Bad
Baghi et al. (74)	L	H	L	L	L	U	L	Good
Dus-Zuchowska et al. (75)	L	L	H	H	L	U	L	Fair
Abedi et al. (76)	L	L	L	L	H	H	L	Fair
Aslani et al. (18)	L	L	H	H	L	U	H	Bad

U, unclear risk of bias; L, low risk of bias; H, high risk of bias.

Good < 2 high risks of bias; Fair = 2 high risks of bias; Bad > 2 high risks of bias.

### Study characteristics

**Figure 2:**
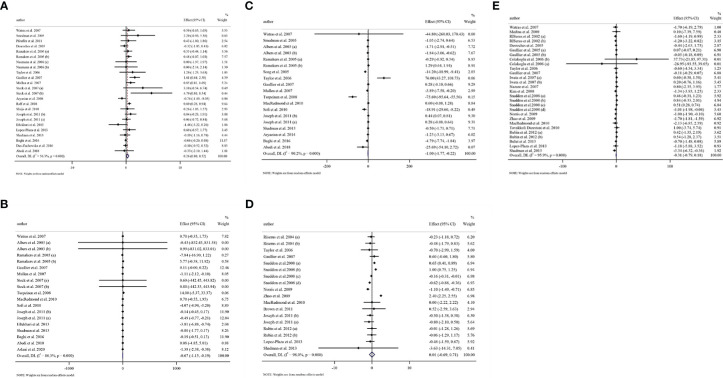
Forest plot detailing weighted mean difference and 95% confidence intervals (CIs) for the effect of CLA consumption on **(A)** CRP (mg/L), **(B)** IL-6 (pg/ml), **(C)** TNF-α (ng/ml), **(D)** adiponectin (µg/ml), and **(E)** leptin (ng/ml). Horizontal lines represent 95% CIs. Diamonds represent pooled estimates from random-effects analysis. WMD, weighted mean difference; CI, confidence interval; CLA, conjugated linoleic acid; CRP, C-reactive protein; IL-6, interleukin 6; TNF-α, tumor necrosis factor-alpha.

All of these studies were RCTs published between 2000 and 2020. Study design characteristics are presented in **Table 1**. The WMD and 95% CI of CRP (mg/dl), IL-6 (pg/ml), TNF-α (ng/ml), adiponectin (µg/ml), and leptin (ng/ml) and their changes are presented in **Figures 2A–E**, respectively. The two studies were conducted in the USA (42, 67), three in Sweden (43, 45, 49), three in the Netherlands (44, 52, 65), four in Canada (12, 14, 46, 68), two in Norway (27, 47), four in the UK (28, 48, 51, 56), two in France (50, 53), two in Turkey (11, 72), one in Japan (55), one in Ireland (57), one in Korean (58), one in Finland (59), one in Denmark (61), one in China (62), three in Germany (62, 69, 70), one in Italy (64), one in Spain (16), one in Poland (75), and the others in Iran (18, 19, 60, 66, 71, 73, 74, 76). Thirteen studies included only men and seven women, and 22 included both sexes. There were 37 parallel (11, 12, 16, 18, 19, 27, 42–45, 47–63, 65–67, 69, 71–76) and five crossover (28, 46, 64, 68, 70) studies. A total of 1,192 subjects (intervention: 597/control: 595) in terms of CRP, 817 subjects (intervention: 418/control: 399) for IL-6, 779 subjects (intervention: 402/control: 377) for TNF-α, 845 subjects (intervention: 424/control: 421) for adiponectin, and 1,274 subjects (intervention: 649/control: 625) for leptin were enrolled. The duration of the intervention varied from 4 to 104 weeks. The CLA supplements were used in doses of 1.6 to 10.8 mg/day. The mean age and baseline BMI ranged from 20 to 61.55 years and 22.2 to 45.71 kg/m^2^, respectively. Participant’s health conditions were as follows: obese men with metabolic syndrome (43), overweight subjects with LDL phenotype B (52), both older obese and young lean, older lean, young obese (28), birch pollen allergy (59), obesity-related hypertension (62), postmenopausal women with type 2 diabetes mellitus (63), overweight mild asthmatics (12), postmenopausal women (66), overweight and hyperlipidemic (68), overweight type 2 diabetes (71), healthy overweight people (16, 47, 56), healthy subjects (11, 42, 44, 48, 49, 51, 53, 54, 57, 58, 61, 64, 67, 70, 72), atherosclerotic patients (73), rheumatoid arthritis (19, 60), athletic (74), overweight/obese (27, 45, 46, 50, 55, 65, 69, 75), nonalcoholic fatty liver disease (76), and chronic obstructive pulmonary disease (COPD) (18). Iwata et al. (55), Albers et al. (44), and Steck et al. used two different CLA doses in their studies. Out of the 42 studies, CRP, IL-6, TNF-α, adiponectin, and leptin were reported in 20, 15, 16, 12, and 20, respectively. For supplementation, 47 study arms used mixed isomers (9cis,11trans-CLA and 10trans,12cis-CLA), four study arms used 9cis,11trans-CLA isomer, and five study arms used 10trans,12cis-CLA isomer. In these studies, detection methods of CRP were Behring latex-enhanced high-sensitivity assays, Eckman Synchron CX7 System, enzyme-linked immunosorbent assay, enhanced turbidimetric immunoassay, highly sensitive immunoassay with a monoclonal antibody coated with polystyrene particles, immunoturbidimetric assay, and rabbit antihuman.

### Meta-analysis findings

#### Effect of CLA supplementation on CRP

Overall, 24 effect sizes from 20 studies for CRP were included in the analysis. The results of the analysis showed that CLA supplementation significantly increased CRP compared to placebo [(WMD = 0.26 mg/dl; 95% CI, 0.00 to 0.52; *p* = 0.048 (*I* ^2^ = 56.3%, <0.001)] (**Figure 2A**). The effect of CLA on CRP was significant in studies with baseline CRP less than 3 mg/dl (WMD = 0.31 mg/dl; 95% CI, 0.05 to 0.57; *p* = 0.016), supplementation dose ≥3 (g/day) (WMD = 0.49 mg/dl; 95% CI, 0.09 to 0.90; *p* = 0.017), and healthy subgroup (WMD = 0.39 mg/dl; 95% CI, 0.10 to 0.68; *p* = 0.008). Heterogeneity disappeared in supplementation subgroups of less than 3 (g/day) (*I* ^2^ = 36.9%, *p* = 0.104), overweight (25–29.9) (*I* ^2^ = 15.9%, *p* = 0.288), and unhealthy subjects (*I* ^2^ = 52.4%, *p* = 0.078). In the other subgroups, the effect of CLA supplementation on serum concentrations of CRP was not significant (**Table 3**).

**Table 3:** Subgroup analyses of CLA supplementation on inflammatory cytokines and adipokines.

	Number of studies	WMD (95%CI)	*p*-value	Heterogeneity	
				*P* heterogeneity	*I* ^2^
Subgroup analyses of CLA supplementation on CRP (mg/dl)					
Overall effect	24	0.26 (0.00, 0.52)	0.048	<0.001	56.3%
Baseline CRP (mg/dl)					
<3	15	0.31 (0.05, 0.57)	0.016	0.003	58.0%
≥3	9	0.27 (−0.71, 1.26)	0.583	0.028	53.4%
Trial duration (week)					
≥12	14	0.44 (−0.01, 0.89)	0.056	0.008	54.3%
<12	10	0.15 (−0.18, 0.49)	0.361	0.007	60.0%
Health status					
Healthy	19	0.39 (0.10, 0.68)	0.008	0.001	57.1%
Unhealthy	5	−0.21 (−0.84, 0.41)	0.508	0.078	52.4%
Supplementation dose (g/day)					
≥3	13	0.49 (0.09, 0.90)	0.017	<0.001	67.3%
<3	11	0.05 (−0.29, 0.40)	0.759	0.104	36.9%
Sex					
Both sexes	16	0.34 (−0.12, 0.81)	0.149	0.009	51.3%
Male	7	0.27 (−0.10, 0.65)	0.151	0.004	68.6%
Female	1	−0.10 (−0.52, 0.32)	0.644	–	–
Baseline BMI (kg/m^2^)					
Normal (18.5–24.9)	3	0.06 (−0.57, 0.71)	0.836	0.001	86.8%
Overweight (25–29.9)	12	0.25 (−0.07, 0.57)	0.131	0.288	15.9%
Obese (>30)	9	0.25 (−0.07, 0.57)	0.111	0.005	63.8%
Isomer’s type					
Both	19	0.25 (−0.05, 0.56)	0.104	<0.001	64.0%
CLA-c9t11	3	0.29 (−0.35, 0.93)	0.373	0.948	0.0%
CLA-t10c12	2	0.45 (−0.08, 0.98)	0.100	0.671	0.0%
Subgroup analyses of CLA supplementation on IL-6 (pg/ml).					
Overall effect	19	−0.66 (−1.14, −0.19)	0.006	<0.001	86.3%
Trial duration (week)					
≥12	10	0.32 (−0.19, 0.83)	0.224	0.335	11.7%
<12	9	−1.16 (−1.82, −0.50)	0.001	<0.001	90.4%
Health status					
Healthy	14	−0.55 (−1.39, 0.28)	0.192	<0.001	87.7%
Unhealthy	5	−0.61 (−1.09, −0.14)	0.010	0.017	67.0%
Supplementation dose (g/day)					
≥3	9	−0.15 (−0.44, 0.14)	0.310	<0.001	71.4%
<3	10	−1.65 (−3.45, 0.14)	0.072	<0.001	79.0%
Sex					
Both sexes	13	−0.84 (−2.09, 0.40)	0.184	<0.001	89.6%
Male	6	−0.31 (−0.51, −0.12)	0.002	0.335	12.5%
Baseline BMI (kg/m^2^)					
Normal (18.5–24.9)	2	−1.66 (−5.15, 1.82)	0.349	0.021	81.1%
Overweight (25–29.9)	10	−0.88 (−2.34, 0.57)	0.236	<0.001	87.4%
Obese (>30)	6	−0.15 (0.49, 0.18)	0.379	0.006	69.5%
Isomer’s type					
Both	17	−0.68 (−1.15, −0.21)	0.005	<0.001	87.2%
CLA-c9t11	1	−7.84 (−16.90, 1.22)	0.090	–	–
CLA-t10c12	1	5.77 (−0.38, 11.92)	0.066	–	–
Subgroup analyses of CLA supplementation on TNF-α (ng/ml)					
Overall effect	19	−0.99 (−1.76, −0.22)	0.011	<0.001	90.2%
Trial duration (week)					
≥12	12	−1.21 (−2.44, 0.02)	0.055	<0.001	92.6%
<12	7	−0.73 (−1.74, 0.27)	0.155	<0.001	82.4%
Health status					
Healthy	15	−2.01 (−3.31, −0.71)	0.002	<0.001	92.1%
Unhealthy	4	0.22 (−0.15, 0.60)	0.238	0.191	36.8%
Supplementation dose (g/day)					
≥3	8	0.09 (−0.53, 0.72)	0.772	<0.001	80.5%
<3	11	−3.08 (−4.91, −1.25)	0.001	<0.001	92.9%
Sex					
Both sexes	13	−1.29 (−2.62, 0.03)	0.056	<0.001	91.8%
Male	6	−1.01 (−1.95, −0.08)	0.033	<0.001	86.7%
Baseline BMI (kg/m^2^)					
Normal (18.5–24.9)	2	−9.29 (−18.51, −0.08)	0.048	0.001	90.7%
Overweight (25–29.9)	11	−0.77 (−1.69, 0.14)	0.099	<0.001	82.1%
Obese (>30)	5	0.35 (−0.35, 1.05)	0.327	<0.001	83.5%
Isomer’s type					
Both	17	−1.41 (−2.31, -0.50)	0.002	<0.001	90.6%
CLA-c9t11	1	−0.29 (−0.92, 0.34)	0.369	–	–
CLA-t10c12	1	1.29 (0.64, 1.93)	<0.001	–	–
Subgroup analyses of CLA supplementation on adiponectin (µg/ml)					
Overall effect	18	0.01 (−0.68, 0.71)	0.976	<0.001	98.0%
Trial duration (week)					
≥12	11	−0.09 (−0.57, 0.39)	0.717	<0.001	93.2%
<12	7	0.20 (−1.39, 1.79)	0.805	<0.001	93.4%
Health status					
Healthy	13	0.05 (−0.38, 0.49)	0.811	<0.001	89.4%
Unhealthy	5	−0.12 (−2.41, 2.17)	0.918	<0.001	98.7%
Supplementation dose (g/day)					
≥3	11	−0.06 (−1.35, 1.23)	0.928	<0.001	97.3%
<3	7	0.13 (−0.46, 0.73)	0.669	<0.001	94.6%
Sex					
Both sexes	6	0.41 (−1.18, 2.00)	0.614	<0.001	88.7%
Male	10	−0.04 (−0.50, 0.42)	0.865	<0.001	92.1%
Female	2	−1.05 (−1.58, −0.52)	<0.001	0.311	2.8%
Baseline BMI (kg/m^2^)					
Normal (18.5–24.9)	3	0.25 (−0.50, 1.01)	0.511	<0.001	93.6%
Overweight (25–29.9)	5	−0.19 (−0.85, 0.47)	0.568	0.943	0.0%
Obese (>30)	10	−0.00 (−1.08, 1.07)	0.995	<0.001	98.5%
Isomer’s type					
Both	15	0.05 (−0.72, 0.82)	0.900	<0.001	98.3%
CLA-c9t11	1	−0.00 (−1.28, 1.26)	0.989	–	–
CLA-t10c12	2	−0.25 (−1.15, 0.63)	0.572	–	90.0%
Subgroup analyses of CLA supplementation on leptin (ng/ml)					
Overall effect	28	−0.30 (−0.79, 0.17)	0.214	<0.001	95.9%
Trial duration (week)					
≥12	19	0.03 (−0.16, 0.23)	0.743	0.002	54.9%
<12	9	−0.81 (−1.71, 0.08)	0.077	0.005	63.8%
Health status					
Healthy	23	0.06 (−0.11, 0.24)	0.463	0.018	42.3%
Unhealthy	5	−1.69 (−1.80, −1.58)	<0.001	0.451	0.0%
Supplementation dose (g/day)					
≥3	19	−0.32 (−0.92, 0.28)	0.297	<0.001	96.8%
<3	9	−0.19 (−0.85, 0.46)	0.567	0.002	67.9%
Sex					
Both sexes	10	−0.64 (−1.47, 0.17)	0.124	<0.001	98.4%
Male	12	0.06 (−0.35, 0.48)	0.754	0.022	50.8%
Female	6	−0.95 (−1.79, −0.12)	0.025	0.696	0.0%
Baseline BMI (kg/m^2^)					
Normal (18.5–24.9)	5	0.50 (0.28, 0.72)	<0.001	0.703	0.0%
Overweight (25–29.9)	14	−0.00 (−0.14, 0.13)	0.966	0.343	10.0%
Obese (>30)	9	−0.73 (−1.59, 0.12)	0.092	<0.001	96.6%
Isomer’s type					
Both	25	−0.33 (−0.85, 0.17)	0.196	<0.001	96.4%
CLA-c9t11	1	0.41 (−1.34, 2.18)	0.643	–	–
CLA-t10c12	2	−0.27 (−1.97, 1.43)	0.756	0.210	36.5%

CI, confidence interval; WMD, weighted mean differences; CLA, conjugated linoleic acid; CRP, c-reactive protein; IL-6, interleukin 6; TNF-α, tumor necrosis factor-alpha.

#### Effect of CLA supplementation on IL-6

Pooling effect sizes from 15 studies showed that CLA supplementation significantly decreased IL-6 compared to placebo [(WMD = −0.66 mg/dl; 95% CI, −1.14 to −0.19; *p* = 0.006 (*I* ^2^ = 86.3%; *p* < 0.001)] (**Figure 2B**). The effect of CLA on IL-6 was significant in trials lasting <12 weeks (WMD = −1.16 pg/ml; 95% CI, −1.82 to −0.50; *p* = 0.001), male subjects (WMD = −0.31 mg/dl; 95% CI, −0.51 to −0.12; *p* = 0.002), unhealthy subgroup (WMD = −0.61 mg/dl; 95% CI, −1.09 to −0.14; *p* = 0.010), and when a mixture of two isomers was used (WMD = −0.68 mg/dl; 95% CI, −1.15 to −0.21; *p* = 0.005). Heterogeneity disappeared in the trial duration of ≥12 week (*I* ^2^ = 11.7%; *p* = 0.335) and the male subgroup (*I* ^2^ = 15.5%; *p* = 0.335). In the other subgroups, the effect of CLA supplementation on serum concentrations of IL-6 was not significant (**Table 3**).

#### Effect of CLA supplementation on TNF-α

Results of pooled 19 random-effects size of CLA supplementation on TNF-α found a significant reduction in TNF-α compared to placebo [(WMD = −0.99 mg/dl; 95% CI, −1.76 to −0.22; *p* = 0.011; (*I* ^2^ = 90.2%; *p* < 0.001)] (**Figure 2C**). Subgroup analysis showed a more beneficial effect of CLA supplementation in studies with an intervention dose of <3 (g/day) (WMD = −3.08 mg/dl; 95% CI, −4.91 to −1.25; *p* = 0.001), healthy people (WMD = −2.01 mg/dl; 95% CI, −3.31 to −0.71; *p* = 0.002), male subjects (WMD = −1.01 mg/dl; 95% CI, −1.95 to −0.08; *p* = 0.033), subjects with normal range of BMI (18.5–24.9) (WMD = −9.29 mg/dl; 95% CI, −18.51 to −0.08; *p* = 0.048), and when a mixture of two isomers was used (WMD = −1.41 mg/dl; 95% CI, −2.31 to −0.50; *p* = 0.002). Interestingly, when the 10trans12cis-CLA isomer was administered, TNF-α was significantly increased (WMD = 1.29 mg/dl; 95% CI, 0.64 to 1.93; *p* < 0.001). Between-study heterogeneity was out of sight in studies with unhealthy subjects (*I* ^2^ = 36.8%; *p* = 0.191). In the other subgroups, the effect of CLA supplementation on serum concentrations of TNF-α was not significant (**Table 3**).

#### Effect of CLA supplementation on adiponectin

Combining 18 effect sizes from 12 studies showed that CLA supplementation had no significant effect on adiponectin level in the intervention group compared to placebo [(WMD = −0.01 mg/dl; 95% CI, −0.68 to 0.71; *p* = 0.976 (*I* ^2^ = 98.0%, *p* < 0.001)] (**Figure 2D**). Subgroup analyses showed a significant decrease in adiponectin levels in the female subjects (WMD = −1.05 mg/dl; 95% CI, −1.58 to −0.52; *p* < 0.001). Our subgroup analyses indicated no significant between-study heterogeneity in studies conducted on the female subjects (*I* ^2^ = 2.8%; *p* = 0.311) and overweight (*I* ^2^ = 0.0%; *p* = 0.943) (**Table 3**).

#### Effect of CLA supplementation on leptin

Combining 28 effect sizes from 20 studies showed that CLA supplementation had no significant effect on leptin level in the intervention compared to the placebo group [(WMD = −0.30 mg/dl; 95% CI, −0.79 to 0.17; *p* = 0.214 (*I* ^2^ = 95.9%; *p* < 0.001)] (**Figure 2E**). Subgroup analyses showed a significant decrease in leptin levels in unhealthy subjects (WMD = −1.69 mg/dl; 95% CI, −1.80 to −1.58; *p* < 0.001), female subjects (WMD = −0.95 mg/dl; 95% CI, −1.79 to −0.12; *p* = 0.025), and a significant increase in leptin levels in the subjects with normal BMI (18.5–24.9) (WMD = 0.50 mg/dl; 95% CI, 0.28 to 0.72; *p* < 0.001). Subgroup analyses indicated no significant between-study heterogeneity in studies conducted on unhealthy subjects (*I* ^2^ = 0.0%; *p*=0.451), female subjects (*I* ^2^ = 0.0%; *p* = 0.696), overweight subjects (*I* ^2^ = 0.0%; *p* = 0.943), and subjects with normal range of BMI (*I* ^2^ = 0.0%; *p* = 0.703) (**Table 3**).

### Publication bias

**Figure 3:**
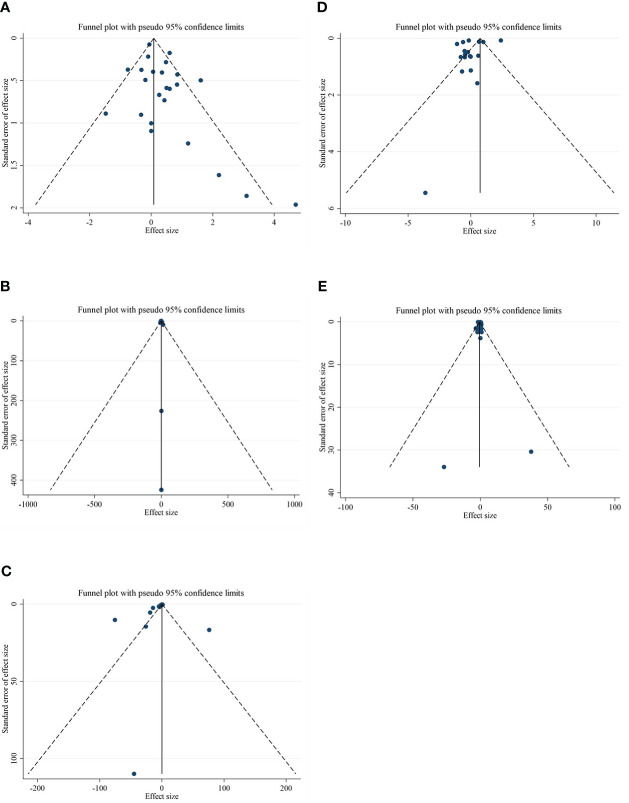
Funnel plots for the effect of CLA consumption on **(A)** CRP (mg/L), **(B)** IL-6 (pg/ml), **(C)** TNF-α (ng/ml), **(D)** adiponectin (µg/ml), and **(E)** leptin (ng/ml). Horizontal lines represent 95% CIs. Diamonds represent pooled estimates from random-effects analysis. WMD, weighted mean difference; CI, confidence interval; CLA, conjugated linoleic acid; CRP, C-reactive protein; IL-6, interleukin 6; TNF-α, tumor necrosis factor-alpha.

Although the visual inspection of funnel plots showed slight asymmetries for all of the outcome variables, no significant publication bias was detected according to Begg’s test for most of the variables, such as CRP (*p* = 0.189), IL-6 (*p* = 0.441), adiponectin (*p* = 0.198), and leptin (*p* = 0.050). Publication bias was considerable just for TNF-α (*p* = 0.042) (**Figures 3A–E**).

### Nonlinear dose-response analysis

**Figure 4:**
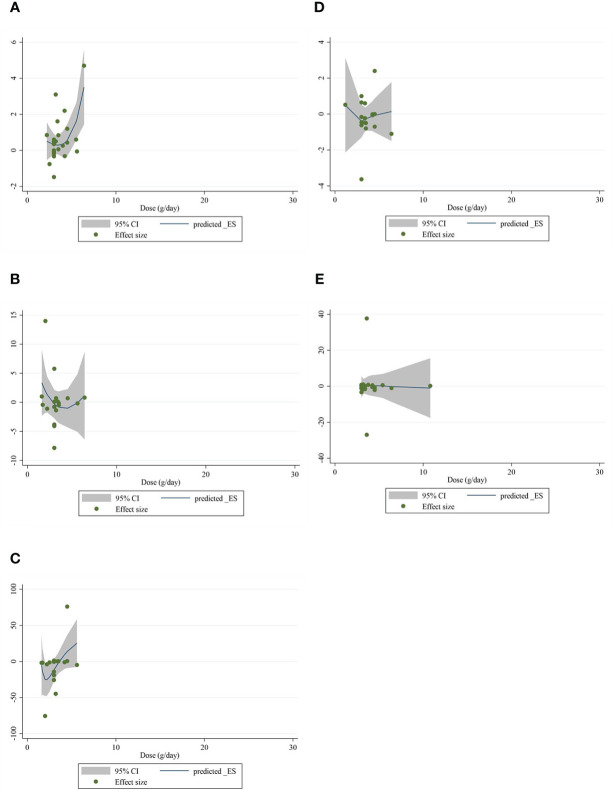
Nonlinear dose–response analysis on the effects of the CLA dosage (mg/day) on **(A)** CRP (mg/L), **(B)** IL-6 (pg/ml), **(C)** TNF-α (ng/ml), **(D)** adiponectin (µg/ml), and **(E)** leptin (ng/ml). Horizontal lines represent 95% CIs. Diamonds represent pooled estimates from random-effects analysis. WMD, weighted mean difference; CI, confidence interval; CLA, conjugated linoleic acid; CRP, C-reactive protein; IL-6, interleukin 6; TNF-α, tumor necrosis factor-alpha.

**Figure 5:**
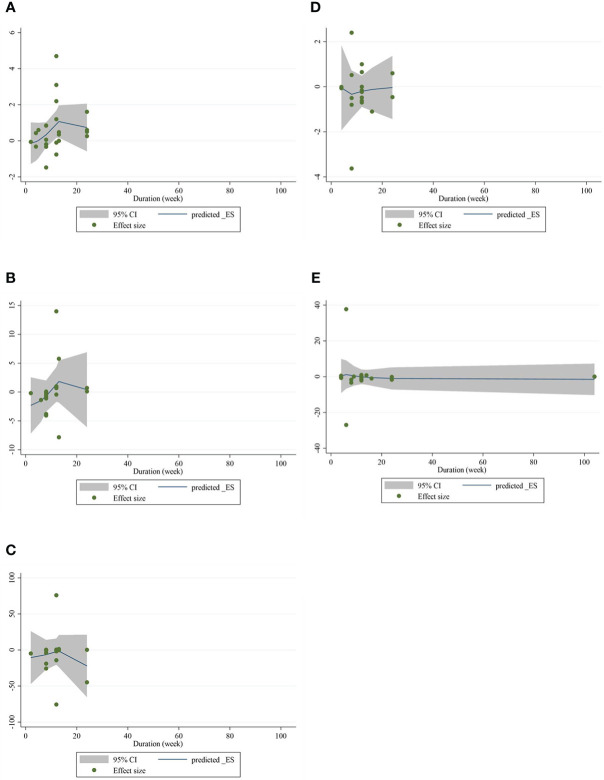
Nonlinear dose–response analysis on the effects of the duration of the intervention (week) of CLA on **(A)** CRP (mg/L), **(B)** IL-6 (pg/ml), **(C)** TNF-α (ng/ml), **(D)** adiponectin (µg/ml), and **(E)** leptin (ng/ml). Horizontal lines represent 95% CIs. Diamonds represent pooled estimates from random-effects analysis. WMD, weighted mean difference; CI, confidence interval; CLA, conjugated linoleic acid; CRP, C-reactive protein; IL-6, interleukin 6; TNF-α, tumor necrosis factor-alpha.

The present study conducted a nonlinear dose–response regression to analyze the dose–response relationship between CLA supplementation and inflammatory cytokines and adipokines. We did not find a significant nonlinear relationship between dose (g/day) and changes in CRP [(coefficients = −0.059; *p*_nonlinearity_ = 0.353)] (**Figure 4A**), IL-6 [(coefficients = −10.437; *p*_nonlinearity_ = 0.244)] (**Figure 4B**), TNF-α [(coefficients = −791.208; *p*_nonlinearity_ = 0.081)] (**Figure 4C**), adiponectin [(coefficients = 1.337; *p*_nonlinearity_ = 0.522)] (**Figure 4D**), and leptin [(coefficients = −77.607; *p*_nonlinearity_ = 0.841)] (**Figure 4E**). Moreover, there was a nonlinear association between the duration of intervention and CRP [(coefficients = −0.798; *p*_nonlinearity_ = 0.164)] (**Figure 5A**), IL-6 [(coefficients = 2.635; *p*_nonlinearity_ = 0.247)] (**Figure 5B**), TNF-α 9[(coefficients = −8.156; *p*_nonlinearity_ = 0.622)] (**Figure 5C**), adiponectin [(coefficients = −0.343; *p*_nonlinearity_ = 0.752)] (**Figure 5D**), and leptin [(coefficients = 0.056; *p*_nonlinearity_ = 0.748)] (**Figure 5E**).

### Meta-regression analysis

**Figure 6:**
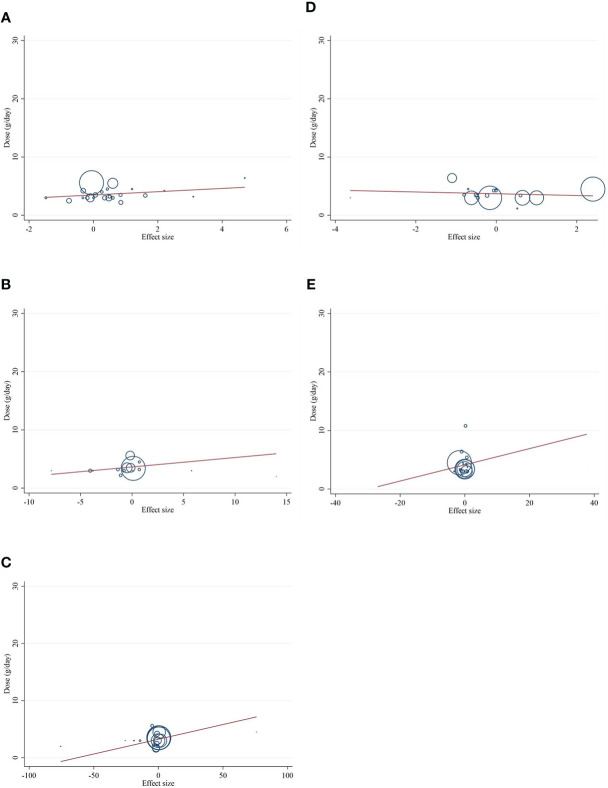
Random-effects meta-regression plots of the association between dose of CLA (mg/day) and weighted mean difference of **(A)** CRP (mg/L), **(B)** IL-6 (pg/ml), **(C)** TNF-α (ng/ml), **(D)** adiponectin (µg/ml), and **(E)** leptin (ng/ml). Horizontal lines represent 95% CIs. Diamonds represent pooled estimates from random-effects analysis. WMD, weighted mean difference; CI, confidence interval; CRP, C-reactive protein; IL-6, interleukin 6; TNF-α, tumor necrosis factor-alpha.

**Figure 7:**
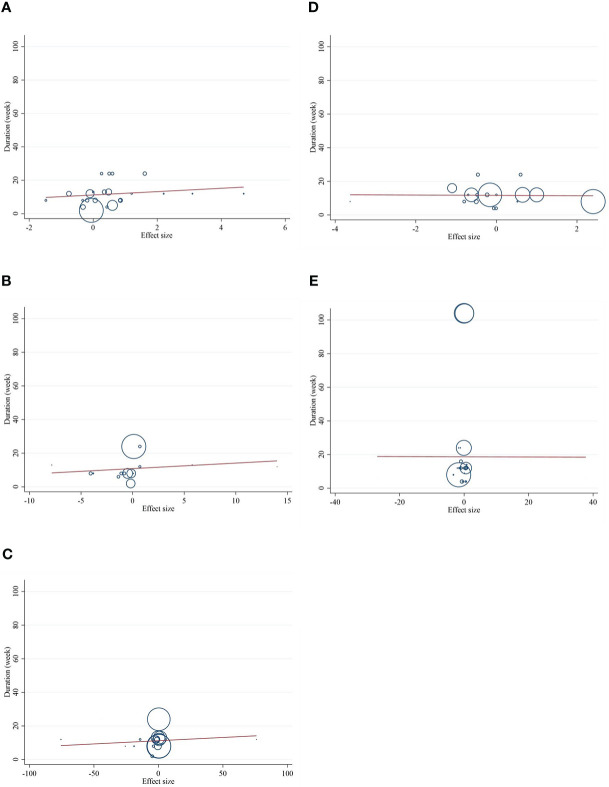
Random-effects meta-regression plots of the association between duration of intervention of CLA and weighted mean difference of **(A)** CRP (mg/L), **(B)** IL-6 (pg/ml), **(C)** TNF-α (ng/ml), **(D)** adiponectin (µg/ml), and **(E)** leptin (ng/ml). Horizontal lines represent 95% CIs. Diamonds represent pooled estimates from random-effects analysis. WMD, weighted mean difference; CI, confidence interval; CRP, C-reactive protein; IL-6, interleukin 6; TNF-α, tumor necrosis factor-alpha.

Meta-regression analyses were performed to assess whether inflammatory cytokines and adipokines were affected by CLA supplementation doses and intervention duration. We found no significant linear association between intervention dose (g/day) and changes in CRP [(coefficients = 0.279; *p*_linearity_ = 0.304)] (**Figure 6A**), IL-6 [(coefficients = 0.162; *p*_linearity_ = 0.384)] (**Figure 6B**), TNF-α [(coefficients = 0.051; *p*_linearity_ = 0.538)] (**Figure 6C** **)**, adiponectin [(coefficients = −0.152; *p*_linearity_ = 0.616)] (**Figure 6D**), leptin [(coefficients = 0.138; *p*_linearity_ = 0.693)] (**Figure 6E**), nor duration of supplementation (week) and changes in CRP [(coefficients = 1.001; *p*_linearity_ = 0.365)] (**Figure 7A**), IL-6 [(coefficients = 0.327; *p*_linearity_ = 0.528)] (**Figure 7B**), TNF-α [(coefficients = 0.038; *p*_linearity_ = 0.737)] (**Figure 7C**), adiponectin [(coefficients = −0.094; *p*_linearity_ = 0.944)] (**Figure 7D**), and leptin [(coefficients = −0.006; *p*_linearity_ = 0.994)] (**Figure 7E**).

### Sensitivity analysis

The sensitivity analysis demonstrated that no study had a significant impact on the overall findings. Although the overall result of studies reporting data on TNF-α was sensitive to the study by Song et al. (48) (WMD = −0.644; 95% CI, −1.353, 0.065), and IL-6 was sensitive to the study by Sofi et al. (64) (WMD = −0.284; 95% CI, −0.601, 0.033), by omitting these studies, the significance of the correlation between CLA and TNF-α and IL-6 was lost.

In the case of CRP, some studies had an impact on the overall effect size, including that of Whigham et al. (54) (WMD = 0.253; 95% CI, −0.013, 0.520), Smedman et al. (49) (WMD = 0.247; 95% CI, −0.010, 0.506), and Pfeuffer et al. (69) (WMD = 0.257; 95% CI, −0.007, 0.523); the arm “a” and “b” of the study of Ramakers et al. (50) (WMD = 0.258; 95% CI, −0.013, 0.529; WMD = 0.245; 95% CI, −0.028, 0.518), Taylor et al. (51) (WMD = 0.251; 95% CI, −0.009, 0.512), Gaullier et al. (27) (WMD = 0.190; 95% CI, −0.053, 0.434), and Mullen et al. (57) (WMD = 0.226; 95% CI, −0.036, 0.490); the arm “a” and “b” of the study of Steck et al. (56) (WMD = 0.245; 95% CI, −0.010, 0.501; WMD = 0.237; 95% CI = −0.011, 0.485), Raff et al. (61) (WMD = 0.219; 95% CI, −0.046, 0.484), and Sluijs et al. (65) (WMD = 0.262; 95% CI, −0.003, 0.529); and the arm “b” of the study of Joseph et al. (68) (WMD = 0.237; 95% CI, 0.026, 0.501) and Lopez-Plaza et al. (16) (WMD = 0.249; 95% CI, −0.016, 0.515). It was shown that the exclusion of every individual study by the sensitivity analysis could not change the direction of the correlation but eliminated the significant effect of CLA on CRP.

### GRADE assessment

**Table 4** displays the Grading of Recommendations Assessment, Development, and Evaluation (GRADE) profile of CLA supplementation on inflammatory cytokine and adipokine variables together with the certainty in outcomes. For CRP, because of serious limitations in inconsistency, the quality of evidence was moderate. In the case of IL-6, because of very serious limitations in inconsistency and for TNF-α because of very serious limitations in inconsistency ad serious limitations for publication bias, the quality of evidence was low. For both outcomes, including leptin and adiponectin, because of very serious limitations in inconsistency and serious limitations in imprecision, the quality of evidence was very low.

**Table 4:** GRADE profile of CLA supplementation for inflammatory cytokines and adipokines.

Outcomes	Risk of bias	Inconsistency	Indirectness	Imprecision	Publication bias	Number of interventions/control	Quality of evidence
CRP	No serious limitation	Serious limitation^a^	No serious limitation	No serious limitation	No serious limitation	1,192 (597/595)	⊕⊕⊕◯ Moderate
IL-6	No serious limitation	Very serious limitation^a^	No serious limitation	No serious limitation	No serious limitation	817 (418/399)	⊕⊕◯◯ Low
TNF-α	No serious limitation	Very serious limitation^a^	No serious limitation	No serious limitation	Serious limitation^c^	779 (402/377)	⊕⊕◯◯ Low
Adiponectin	No serious limitation	Very serious limitation^a^	No serious limitation	Serious limitation^b^	No serious limitation	845 (424/421)	⊕◯◯◯ Very low
Leptin	No serious limitation	Very serious limitation^a^	No serious limitation	Serious limitation^b^	No serious limitation	1,274 (649/625)	⊕◯◯◯ Very low

CLA, conjugated linoleic acid; CRP, c-reactive protein; IL-6, interleukin 6; TNF-α, tumor necrosis factor-alpha.

a There is a significant heterogeneity for CRP (I^2^ = 56.3%%), IL-6 (I^2^ = 86.3%), TNF-α (I^2^ = 90.2%), adiponectin (I^2^ = 98.0%), and leptin (I^2^ = 95.9%).

b There is no evidence of significant effects of CLA supplementation on adiponectin and leptin.

c There is significant publication bias for TNF-α (p = 0.042).

### Certainty assessment

Using the GRADE methodology, which was previously outlined, the total degree of evidence certainty across the studies was evaluated and summarized (77).

## Discussion

In this review, an analysis of pooling 42 studies indicated that CLA supplementation increased CRP concentrations, decreased IL-6 and TNF-α values, and had no effect on adiponectin and leptin levels. Findings of subgroup analyses showed that CLA consumption increased CRP in subjects with normal CRP levels (less than 3 mg/dl) and those who were healthy or consumed CLA in doses of ≥3 g/day. Taking CLA decreased IL-6 in male individuals and unhealthy subjects if the trial duration was less than 12 weeks and when a mixture of two isomers was used. TNF-α was reduced by CLA consumption in studies with an intervention dose of <3 g/day, male subjects, healthy persons, or those having normal baseline BMI, and when a mixture of two isomers was used. However, in subgroup analyses based on isomer type, 10trans12cis- CLA isomer significantly increased TNF-α. Supplementation with CLA reduced adiponectin in women. It also decreased leptin in women and unhealthy adults but increased in those with normal baseline BMI. Moreover, the results of the meta-regression analysis showed that there was no significant linear association between intervention doses and duration of supplementation with inflammatory cytokines and adipokines.

The majority of studies included in this meta-analysis used supplements consisting of mixed amounts of CLA isomers. Therefore, since CLA isomers have shown different effects on inflammation (30, 78), it is suggested that this issue be considered in research. It is also important to consider the contents of the placebo used in studies. Some included RCTs have used vegetable oils as a placebo, such as safflower and olive oils. One study has indicated the reduced effect of safflower oil on CRP levels compared to CLA (79). Another study has elucidated the anti-inflammatory effect of olive oils through the prevention of lipooxygenase and enzymes that synthesize leukotriene (80). In other words, prescribing these vegetable oils as a placebo might misinterpret results.

Diet and physical activity can play an important role in inflammatory conditions (81). It is better to consider the effects of these two factors in the result of the present meta-analysis. Although, in some studies, the confounding variables of physical activity and diet were controlled, in some trials, these factors were not considered.

Diet is an important regulatory factor in the immune response. Malnutrition leads to suppression of the immune system, whereas overnutrition leads to immune activation. Some foods have anti-inflammatory effects, and there are still controversies about others (81). For example, several studies have reported a positive association between the glycemic index/glycemic load of dietary carbohydrates and inflammatory cytokines (81). Also, a high-fat diet causes excessive body fat accumulation and impairs the immune system. On the other hand, it has been reported that trans and saturated fatty acids are significantly associated with the inflammatory state (82). Although ruminant trans fatty acids have different effects than industrial trans fatty acids, several studies show that industrial trans fatty acids promote inflammation, whereas ruminant trans fatty acids have been reported to be harmless or even beneficial to health, as well as also having anti-inflammatory properties (83).

In relation to physical activity, it has been reported that although exercise produces a short-term inflammatory response, regular exercise in the long term has an “anti-inflammatory” effect (84). One study reported that sedentary subjects had higher levels of inflammatory factors such as IL-6 and TNF-α compared to subjects with higher physical activity (85).

Other confounding factors that can affect inflammatory markers are sleep duration and sleep quality. Recently, studies have reported a relationship between longer sleep duration (>8 h) and increased levels of inflammatory markers such as CRP and IL-6 (86, 87). In addition, a study on healthy subjects reported that higher IL-6 levels were associated with lower sleep quality (88).

Here, we showed CLA consumption increased CRP concentrations, decreased IL-6 and TNF-α, and did not change adiponectin and leptin levels. There are some meta-analyses that have investigated the effect of CLA on the abovementioned markers. For example, Derakhshandeh-Rishehri et al. (2019) (89), working on 15 RCTs, revealed that long-term (24 weeks) CLA intake significantly increased CRP levels (89). A meta-analysis conducted by Haghighatdoost et al. (28) showed a significant increase in CRP (*n* = 8) and TNF-α levels (*n* = 8) along with a slight decrease in blood IL-6 (*n* = 9) following CLA supplementation (29). Another meta-analysis (2018) conducted on 19 RCTs demonstrated that CLA intake had a small but not significant decline in plasma leptin. However, it significantly decreased leptin in obese individuals, with trials lasting for less than 24 weeks (21). A study carried out by Mazidi et al. (89) on 14 RCTs indicated that treatment with CLA had no effect on IL-6 but significantly reduced serum TNF-α and adiponectin, besides enhancing serum CRP concentrations (90). By combining previous and current results, it can be acknowledged that except for the effect of CLA in increasing CRP levels, there is no consensus on other markers (i.e., IL-6, TNF-α, adiponectin, and leptin). Moreover, it is noteworthy to state that exercise, dietary intake, sleep duration, and sleep quality can alter the effects of CLA. Therefore, further well-designed studies may clarify the role of CLA on the abovementioned markers.

In the present study, the gender-dependent impact of CLA has also been shown in the reduction of IL-6, TNF-α, adiponectin, and leptin. Based on evidence, CLA can induce testosterone biosynthesis (91), and subsequently, testosterone is able to decrease IL-6 expression (92). It has also been stated that circulating CLA concentrations are greater in women than in men (93). Therefore, the aforementioned evidence can justify the different effects seen between men (IL-6 and TNF-α reduction) and women (adiponectin and leptin reduction) after taking CLA.

The effect of CLA on inflammatory responses has generated inconsistent results. This may be due to isomers and tissue-specific responses of CLA. Moreover, across the included RCTs, administration doses of CLA varied from 1.6 to 10.8 mg/day, and the duration of the treatment differed from 4 to 104 weeks. Health characteristics, inflammation status, and even gut microbiome composition of the recruited individuals were also different (78). These variations in the studies can influence the results and interpretations.

For example, in relation to isomer and tissue-specific response, 9cis,11trans-CLA isomer exerts its anti-inflammatory effect by activating the peroxisome proliferator-activated receptor-γ (PPAR-γ)-dependent pathway and ultimately reducing the production of proinflammatory cytokines such as TNF-α and IL-6 (30). However, the 10trans,12cis-CLA isomer has been reported to have a proinflammatory effect in adipose tissue, in contrast to its effects on innate immunity and vascular cells. In addition, in adipose tissue, activation of PPAR­γ is contrary to the physiological effects of 10trans,12cis-CLA *in vitro* and *in vivo* (78). This isomer downregulates PPAR-γ gene expression and increases TNF-α, IL-6, and CRP production in adipose tissue (78, 94). It seems that the reason for this inconsistency is cytokines secreted from adipocytes, which in turn alter PPAR­γ expression and activity in the fat cell. Therefore, inconclusive results regarding the anti-inflammatory properties of CLA are likely due to the subtle proinflammatory effects of the 10trans,12cis-CLA isomers in specific tissues (30, 78). Overall, it appears that CLA can elicit both anti-inflammatory and proinflammatory features.

Some proposed mechanisms for the effects of CLA isomers on inflammation include the following: (1) Modulation of eicosanoid signaling. This means that CLA reduces the production of inflammatory eicosanoids derived from arachidonic acid (AA) through the inhibition of several steps in the AA cascade. (2) Modulating the transcription of some genes, such as the PPAR gene, plays an important role in the regulation of inflammation. Also, it suppresses the expression of the inducible NO synthase (iNOS) gene, which leads to a decrease in IL-6 production. (3) Inhibition of NF-kB activity, which is a transcription factor involved in cytokine gene expression, cellular adhesion, cell cycle activation, apoptosis, and carcinogenesis and is integrally related to inflammatory responses (78, 95).

This meta-analysis has some limitations that should be addressed. Most of the included studies had a relatively small sample size, which can cause an overestimation of the results. Observation of publication bias for TNF-α findings suggests overestimation of CLA efficiency on TNF-α. Moreover, TNF-α, IL-6, and CRP results were sensitive to some studies.

In conclusion, it is suggested that CLA supplementation can have both proinflammatory and anti-inflammatory roles. It can enhance CRP concentrations while reducing TNF-α and IL-6 levels. Furthermore, CLA is able to decrease adiponectin and leptin in women. It can also decrease leptin in unhealthy adults and increase it in subjects with a normal baseline BMI. Finally, in order to improve the quality of studies, future clinical trials are encouraged to carefully consider CLA-isomer-specific regulation of inflammatory markers and take notice of the contents of the placebo used in their control groups. It is also important to keep in mind the gender-dependent impact of CLA.

## Data availability statement

The original contributions presented in the study are included in the article/supplementary material. Further inquiries can be directed to the corresponding authors.

## Author contributions

OA and SR contributed to the conception and design of the study. DA-L, MZ, and GS contributed to data extraction. FS and AK screened articles for inclusion criteria. OA contributed to the data analysis. SR, MY, and EG contributed to manuscript drafting, OA and MZ supervised the study. All authors contributed to the article and approved the submitted version.
